# Case report: Breaking CNS immuno-privilege: TNFα-inhibitor triggers aseptic meningitis in a patient with rheumatoid arthritis

**DOI:** 10.3389/fimmu.2024.1432360

**Published:** 2024-09-10

**Authors:** Benedetta Kassabian, Monica Facco, Alessandro Miscioscia, Samuela Carraro, Francesca Rinaldi, Paolo Gallo, Marco Puthenparampil

**Affiliations:** ^1^ Neurology Unit, Department of Neuroscience (DNS), Università degli Studi di Padova, Padua, Italy; ^2^ Laboratory of Hematology and Immunology, Department of Medicine (DIMED), Università degli Studi di Padova, Padua, Italy; ^3^ Multiple Sclerosis Centre, Neurology Unit, Azienda Ospedaliera di Padova, Padua, Italy

**Keywords:** rheumatoid arteritis, etanercept, aseptic meningitis, blood-brain barrier, cerebrospinal fluid, PET-MRI

## Abstract

Blood-brain barrier dysfunction might be driven by peripheral inflammation. TNFα inhibitors (TNF-α_i_) are occasionally associated with a wide spectrum of neurological immuno-mediated disorders. However, patients with systemic autoimmune disorders, including rheumatoid arthritis (RA), might be prone to develop further organ-specific, including central nervous system (CNS), autoimmunity. Here we report the case of a patient, affected by RA and treated with etanercept, who suddenly developed focal neurological symptoms. Cerebrospinal fluid, magnetic resonance imaging (MRI), and positron emission tomography (PET)/MRI findings are reported and support the diagnosis of TNF-α_i_ -associated aseptic meningitis.

## Introduction

Peripheral inflammation might induce blood-brain barrier (BBB) dysfunction, breaking the central nervous system (CNS) immune privilege and inducing local inflammation (1). Indeed, TNFα inhibitors (TNF-α_i_) are occasionally associated with a wide spectrum of neurological immuno-mediated disorders (2) that are commonly divided into demyelinating (3–8) and non-demyelinating (9–15). Multiple sclerosis (MS), radiologically isolated syndrome (16), transverse myelitis, and neuromyelitis optica spectrum disorder (NMOSD) are commonly included in demyelinating disorders, while neurosarcoidosis, CNS vasculitis, leptomeningitis, or meningoencephalitis define the non-demyelinating CNS events.

Conversely, patients with systemic autoimmune disorders, including rheumatoid arthritis (RA), might be prone to develop further organ-specific, including CNS and meningeal, autoimmunity (15). Therefore, the presence of CNS involvement in a systemic autoimmune disorder treated with TNF-α_i_ constitutes a diagnostic challenge for physicians. Here we report the case of a patient, affected by RA and treated with *etanercept*, who developed aseptic meningitis.

## Case report

**Figure 1:**
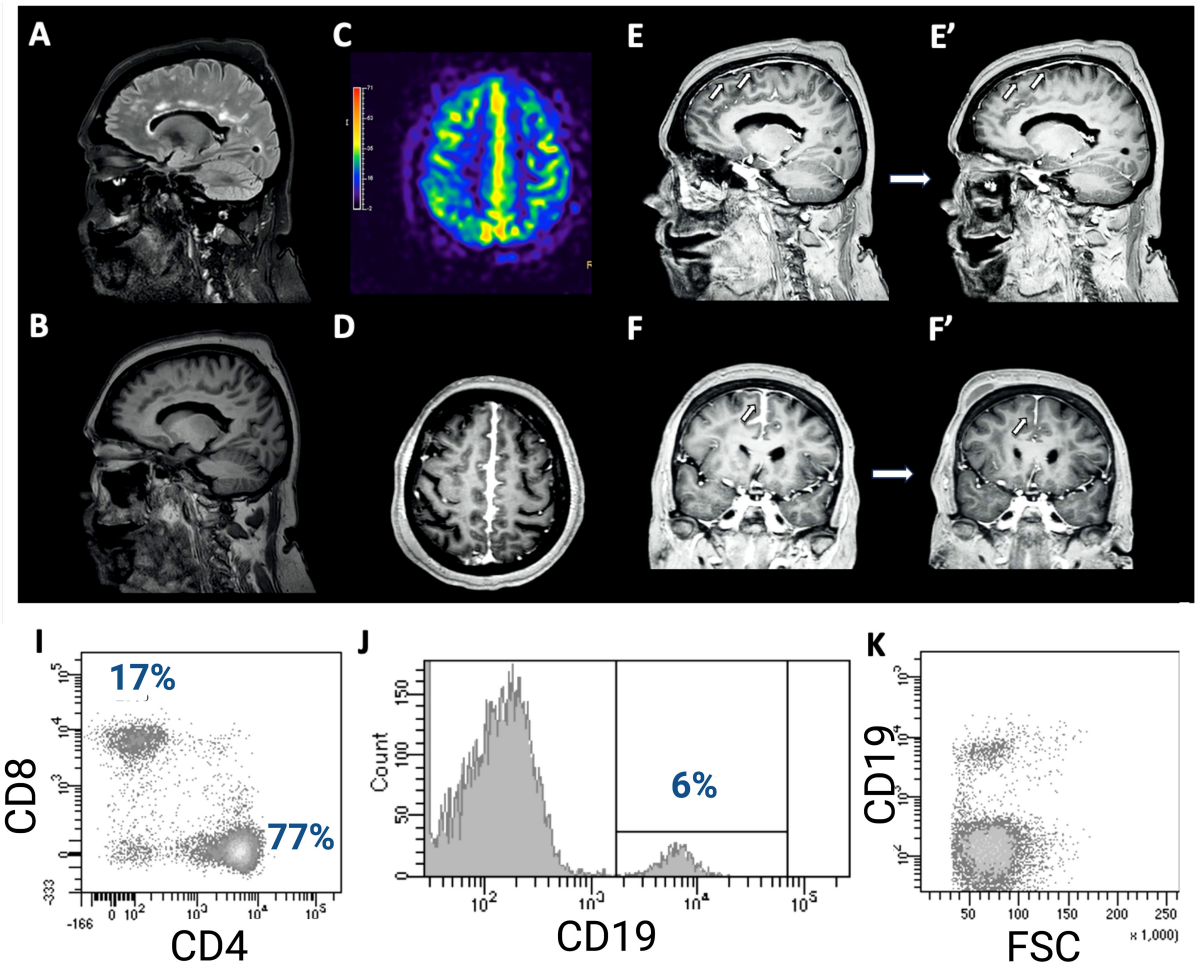
**(A)** Brain MRI showing a slight T2-FLAIR hyperintensity of the cortical gyri in the fronto-parietal area at the vertex, in the parasagittal area, with obliteration of the sulci. Comparing T1 sequences **(B)** and contrast-enhanced T1 sequences **(D–F)**, there is a noticeable pathological pachymeningeal and leptomeningeal enhancement along the profile of the cerebral falx, especially in the posterior region, further confirmed by PET/MRI **(C)**. In addition, a diffuse slight dural meningeal thickening was also reported. Follow-up brain MRI 4 weeks later shows significant improvement of the meningeal enhancement **(E’, F’)**. The total body PET/MRI confirmed the cortical hypermetabolism along the falx cerebri, showing also a slightly hypometabolic left parietal cortex **(E)**. Flowcytometry analysis of CSF-infiltrating cells revealed both CD4+ and CD8+ T-cells **(I)** with an increased ratio (4.5), as well as a small percentage of B cells (6%, **(J)**), with normal physical parameters **(K)**.

A 68-year-old woman was evaluated for a sudden onset transient paresis of the left lower limb lasting for 15 minutes. She reported a few similar spells before, always very brief, occasionally involving the contralateral limb. She was diagnosed with RA 2 years before, without any evidence of either peripheral nervous system (PNS) or CNS involvement. She was temporarily treated with acetylsalicylic acid for the neuroradiologic evidence of microvascular ischemic disease, which was discontinued after spontaneous ecchymosis. Ongoing disease-modifying drugs included low-dose prednisone, etanercept (28 infusions, the last 1 week before the onset of symptoms), and methotrexate. Moreover, she took alendronate for osteoporosis, venlafaxine for depression, and beta-blocker for tachycardia. Neurological examination was normal, while a brain CT scan revealed a slight disappearance of parafalcial cortical sulci bilaterally, confirmed by a high field (3T) magnetic resonance imaging (MRI) scan (**Figure 1**). In addition, a high field (3T) positron emission tomography (PET)/MRI confirmed the cortical hypermetabolism along the falx cerebri (**Figure 1E**). Altogether, brain imaging revealed an inflammation along pachy- and leptomeningeal sheets.

The etiology of this process was investigated with cerebrospinal fluid (CSF) analysis, which disclosed a moderate pleocytosis (125 cells/μL, all mononuclear cells) with a mild increase in protein concentration (54 mg/dL) and CSF/serum quotient of albumin (Q_ALB_) (11.2 x10^-3^, Q_ALB_/Q_LIM_: 1.31, mild BBB damage) (1). Moreover, while isoelectric focusing did not disclose any IgG oligoclonal band (IgGOB), quantitative parameters of intrathecal IgG synthesis were increased (IgG Index 1.2, nv <0.7; IgG_LOC_ 21.0 mg/L). CSF microbiological screening (Streptococcus agalactiae, Streptococcus pneumoniae, Cryptococcus neoformans, cytomegalovirus, enterovirus, herpes simplex virus 1, herpes simplex virus 2, herpes simplex virus 6, human parechovirus, varicella zoster virus, M. tuberculosis cDNA, E. coli K1, Haemophilus influenzae, Lysteria monocytogenes, Neisseria meningitidis) was negative, while the immunophenotype of CSF-infiltrating leucocyte revealed that CSF-infiltrating cells were all lymphocytes, namely both B (6%) and T cells (94%, 77% CD4^+^, 17% CD8^+^) (**Supplementary Figure**). No T cell had increased HLA-DR expression. Physical parameters and the lack of HLA-DR expression suggested the recruitment of inactivated B cells. Immunological screening in serum revealed an elevated rheuma-test (153 kU/L, nv <30), while C-reactive protein, angiotensin-converting enzyme (also in CSF), complement factors, and erythrocyte sedimentation rate were normal. Moreover, anti-nuclear, anti-neutrophilic cytoplasmic, anti-double-stranded DNA, anti-extractable nuclear antigen, anti-anticardiolipin, and β_2_-glycoproteinI antibodies were negative, as well as neoplastic and paraneoplastic markers. Therefore, these findings were strongly consistent with an immuno-mediated CNS-restricted inflammation. Since there were neither systemic symptoms nor serological tests suggestive for RA worsening or progression, TNF-α_i_ was considered as the trigger of this picture. Therefore, said therapy was discontinued, and the patient was treated with methylprednisolone 1 g daily for 5 days, then slowly tapered with oral prednisone. The 1-month follow-up MRI showed radiological improvement with significant resolution of the abnormal meningeal enhancement. The patient did not experience any further spells in the following 6 months.

## Discussion

Our patient reported transitory neurological deficit, characterized by left limb weakness. To evaluate the ischemic etiology, brain imaging was acquired, revealing a severe and diffuse inflammatory meningeal involvement, further confirmed by standard CSF analysis (moderate pleocytosis). Although the clinical symptom could not be considered as a typical or atypical clinically isolated syndrome (17, 18), the MRI picture also disclosed white matter lesion around ventricles, which, especially in presence of an ongoing TNF-α_i_, called for a carefully exclusion of any demyelinating disorder. Since white matter lesion etiology is wide, and in presence of a putative radiologically isolated syndrome, the evaluation of their characteristic is strongly recommended to increase the specificity of criteria for MS diagnosis (19). In our case, the patient presented periventricular lesions, which were not suggestive for MS. Indeed, callosal lesions touched the top of the *corpus callosum*, not the bottom, suggesting an arteriolar pathology rather than a perivenular inflammation (20, 21). In addition, the radiological scenario was characterized by diffuse meningeal inflammation, which is not suggestive of MS. Finally, the dissemination in space criterion was not met in the absence of any cortical-juxtacortical or infratentorial or spinal cord lesion. In addition, the absence of IgGOB or any contrast-enhancing lesions did not allow us to fulfill the dissemination in time criterion. Therefore, a better explanation was more convincing (18).

CSF microbiological screening excluded an infective origin of the severe meningeal inflammation, while total body PET/MRI did not identify any malignancy, ruling out meningeal carcinomatosis.

Therefore, the inflammatory etiology seemed to be more plausible. To distinguish TNF-α_i_ aseptic meningitis from rheumatoid meningitis the evaluation of serological markers, as well as of clinical symptoms, is mandatory.

In our patient, the lack of any clinical or biochemical sign of disease worsening (i.e., the absence of systemic involvement and the normal immunological screening, except for a mild increase of rheumatoid factor), together with the absence of relevant BBB damage suggested an aseptic meningeal involvement.

Indeed, meningeal involvement as an expression of RA (rheumatoid meningitis) is a rare condition that normally presents with an increase in acute phase reactants and in anticitrullinated protein antibodies, that we did not find in this case (22–29). Notably, two case of rheumatoid meningitis were treated with etanercept (24, 26). However, a few cases of RA developed aseptic meningitis when treated with infliximab (14, 30), etanercept (31), and adalimumab (32, 33), suggesting that it is an extremely rare TNF-α_i_ -induced adverse event (15).

From a clinical point of view, the epileptic etiology of the transient symptoms was excluded, since the electroencephalography showed short sequences of slow bitemporal waves that were not consistent with either atonic seizures (high voltage slow wave) or focal negative motor seizures (involving the motor area). Ultimately, the most reliable cause of these stroke-like episodes seems to be the involvement of cortical vessel walls by the leptomeningeal inflammation. From an immunological point of view, the absence of activated B and T cells in CSF, as well as the lack of CSF-restricted IgGOB and of any parenchymal lesion, in the presence of the BBB damage (increased Q_ALB_) that we observed in our patient seems to drive a bystander recruitment of lymphocytes, questioning the presence of a specific CNS-derived-antigen and suggesting a TNF-α_i_-induced innate-immunity disorder. The link between TNF-α_i_ and BBB function warrants further investigation.

## Data Availability

The original contributions presented in the study are included in the article/**Supplementary Material**. Further inquiries can be directed to the corresponding author.
