# Supplementary figures and images for: Case report: Breaking CNS immuno-privilege: TNFα-inhibitor triggers aseptic meningitis in a patient with rheumatoid arthritis
Source: Front Immunol. 2024 Sep 10;15:1432360. doi: 10.3389/fimmu.2024.1432360 (PMC11420026; doi:10.3389/fimmu.2024.1432360)

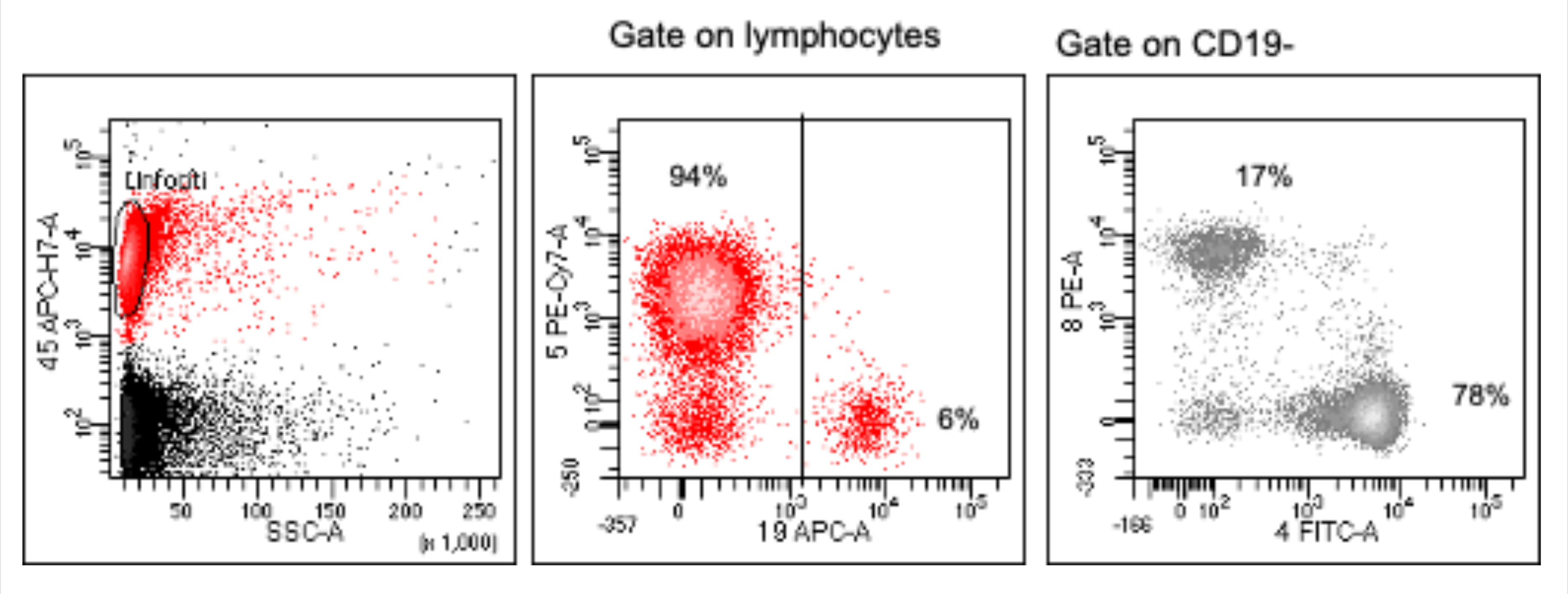

Supplement: Supplementary file 1 [file Image1.jpeg]
